# Requirement for CCNB1 in mouse spermatogenesis

**DOI:** 10.1038/cddis.2017.555

**Published:** 2017-10-26

**Authors:** Ji-Xin Tang, Jian Li, Jin-Mei Cheng, Bian Hu, Tie-Cheng Sun, Xiao-Yu Li, Aalia Batool, Zhi-Peng Wang, Xiu-Xia Wang, Shou-Long Deng, Yan Zhang, Su-Ren Chen, Xingxu Huang, Yi-Xun Liu

**Affiliations:** 1 State Key Laboratory of Stem Cell and Reproductive Biology, Institute of Zoology, Chinese Academy of Sciences, Beijing 100101, China; 2 University of Chinese Academy of Sciences, Beijing 100049, China; 3 School of Life Science and Technology, Shanghai Tech University, 100 Haike Road, Pudong New Area, Shanghai 201210, China; 4 MOE Key Laboratory of Model Animal for Disease Study, Model Animal Research Center of Nanjing University, Nanjing 210061, China

## Abstract

Spermatogenesis, which involves mitosis and meiosis of male germ cells, is a highly complicated and coordinately ordered process. Cyclin B1 (CCNB1), an important regulator in cell cycle machinery, is proved essential for mouse embryonic development. However, the role of CCNB1 in mammalian spermatogenesis remains unclear. Here we tested the requirement for CCNB1 using conditional knockout mice lacking CCNB1 in male germ cells. We found that ablation of CCNB1 in gonocytes and spermatogonia led to mouse sterile caused by the male germ cells’ depletion. Gonocyte and spermatogonia without CCNB1 is unable to proliferate normally and apoptosis increased. Moreover, CCNB1 ablation in spermatogonia may promote their differentiation by downregulating *Lin28a* and upregulating let-7 miRNA. However, ablation of CCNB1 in premeiotic male germ cells did not have an effect on meiosis of spermatocytes and male fertility, suggesting that CCNB1 may be dispensable for meiosis of spermatocytes. Collectively, these results indicate that CCNB1 is critically required for the proliferation of gonocytes and spermatogonia but may be redundant in meiosis of spermatocytes in mouse spermatogenesis.

In eukaryotic cells, the onset of M phase is controlled by a common mechanism. Maturation-promoting factor or M-phase-promoting factor (MPF),^1, 2, 3, 4, 5, 6, 7, 8, 9^ which is composed by cyclin-dependent kinase 1 (CDK1) and cyclin B, governs M-phase entry in eukaryotic cells.^10, 11, 12, 13, 14, 15, 16, 17^ The activation of MPF requires the dephosphorylation of CDK1 and the association of Cyclin B.^18, 19, 20, 21, 22, 23^ In amphibian, two B-type cyclins, cyclin B1 (encoded by *ccnb1*) and cyclin B2 (encoded by *ccnb2*), have been reported to associate with Cdk1 and promote MPF activation.^11, 24^ In frog oocytes, microinjection of *ccnb1* or *ccnb2* mRNA into immature oocytes could induce germinal vesicle breakdown (GVBD); co-inhibition of *ccnb1* and *ccnb2* endogenous mRNA translation, but not one of them, with antisense RNAs can inhibit progesterone-induced GVBD.^24^ Moreover, in the extracts of active *Xenopus* eggs, ablation of either *ccnb1* or *ccnb2* alone was unable to arrest mitosis, but when both cyclin mRNAs were destroyed, the mitosis events were unable to happen.^11^ These results suggest that *ccnb1* and *ccnb2* have redundant roles in the mitosis and meiosis of frog.

However, CCNB1 and CCNB2 were reported to have different localization and expression pattern in mammals,^25, 26, 27, 28^ indicating that they may have distinct roles in mitosis and meiosis of mammalian cells. In human tissue cultured cells, both CCNB1 and CCNB2 are associated with CDK1 and promote CDK1 activation during mitosis; but their localized structure in cell is quite different: CCNB1 to microtubules, CCNB2 primarily to Golgi apparatus.^25, 26^ In mouse testis, *Ccnb2* mRNAs were found primarily in mitotically dividing spermatocytes, whereas *Ccnb1* transcripts were most abundant in the postmeiotic germ cells.^27, 28^

In addition, *Ccnb1*-null mice die *in utero*, whereas *Ccnb2*-null mice is viable and fertile,^29^ suggesting that CCNB1 is critically required for mouse embryogenesis, whereas CCNB2 is largely redundant in mouse embryogenesis and productivity. However, whether CCNB1 and CCNB2 have distinct roles in mitosis and meiosis of mammalian cells remain unclear.

Mouse spermatogenesis, which involves mitosis of spermatogonia and meiosis of spermatocytes, is a powerful *in vivo* system to study the regulation of mitosis and meiosis in mammals. In the present study, we generated conditional knockout mice lacking CCNB1 in different stages of male germ cells to shedding light on the function of CCNB1 in mitosis and meiosis of male germ cells. We found that CCNB1 was critically required for the proliferation of gonocytes and spermatogonia. These cells lacking CCNB1 were unable to proliferate normally and apoptosis increased. We also found that ablation of CCNB1 in spermatogonia might promote their differentiation by downregulating *Lin28a* and upregulating let-7 miRNAs. However, deletion of *Ccnb1* in postnatal, premeiotic male germ cells did not have an effect on spermatocyte meiosis and male fertility, suggesting that CCNB1 may be redundant in meiosis of spermatocytes.

## Results

### Generation of conditional knockout mice lacking CCNB1 in male germ cells

**Figure 1:**
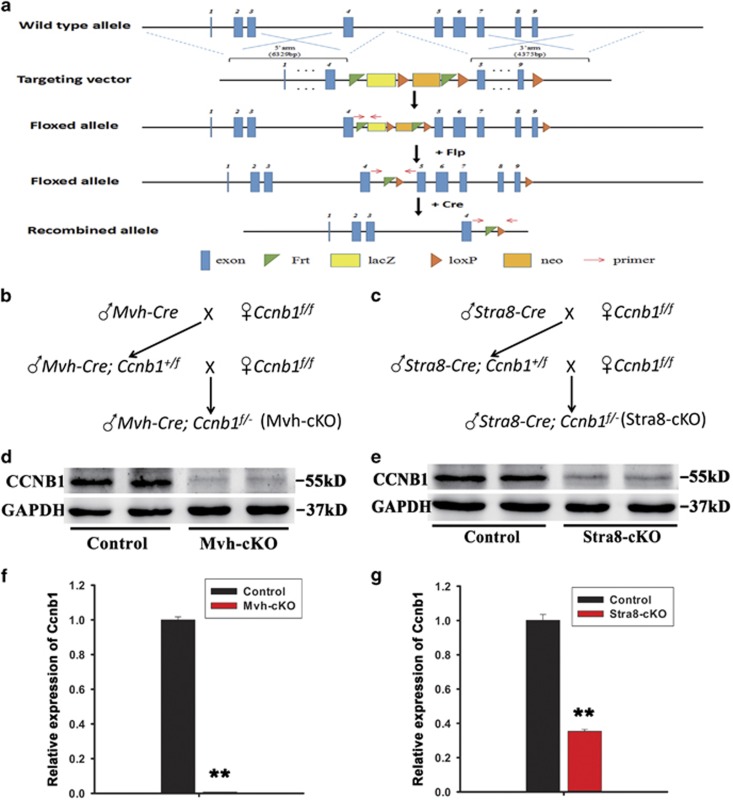
Generation of *Ccnb1* conditional knockout mice. (**a**) The target strategy of Ccnb1. LoxP site were inserted behind exons 4 and 9 of *Ccnb1* allele; when the floxed allele crossed with Cre, exons 5–9 were deleted. (**b**) Mating strategy to generate all germ cell conditional knockout *Ccnb1* mice (Mvh-cKO). (**c**) Mating strategy to generate postnatal, premeiotic male germ cell conditional knockout *Ccnb1* mice (Sta8-cKO). (**d**) Western blotting analysis of Ccnb1 in adult (8–12 weeks) Mvh-cKO and Control littermates’ testis. (**e**) Western blotting analysis of Ccnb1 in adult Stra8-cKO and Control littermates’ testis. (**f**) Real-time PCR analysis of Ccnb1 expression in adult Control and Mvh-cKO mice testis. (**g**) Real-time PCR analysis of Ccnb1 expression in adult Control and Stra8-cKO mice testis. In panels (**f** and **g**), ≥3 samples were used in each group in qPCR

In order to test the requirement for CCNB1 function in male germ cell mitosis and meiosis, we generated two strains of mice, one specifically lacking CCNB1 in all male germ cells and the other lacking CCNB1 in postnatal, premeiotic male germ cells. An ES cell line (Clone No. EPD0357_2_A11) from EUCOMM with *Ccnb1* gene targeting was used for microinjection to generate the mouse model. To achieve the *Ccnb1* gene targeting, an L1L2_Bact_P cassette was inserted at position 100782436 of Chromosome 13 between exons 4 and 5. The cassette, which is flanked by two FRT sites, is composed of lacZ sequence, the first loxP site, neomycin under the control of the human beta-actin promoter and SV40 poly A. The cassette end with the second loxP site and the third loxP site is inserted downstream of the targeted exon 9 at position 100779501. A ‘conditional ready’ (floxed) allele can be created by flp recombinase expression, leaving exons from 5 to 9 flanked by loxP sites (Figure 1a). We then crossed mice homozygous for the floxed *Ccnb1* allele (*Ccnb1*^*f/f*^) with *Mvh-*Cre or *Stra8-*Cre transgenic mice, which express Cre recombinase under the Mvh promoter and Stra8 promoter, respectively (Figures 1b and c).^30, 31^ The mRNA and protein level of *Ccnb1* were tested in the adult mice testes by QRT-PCR and western blotting, respectively. Both protein (Figures 1d and e) and mRNA (Figures 1f and g, numbers of samples used in each group were ≥3) of *Ccnb1* in *Ccnb1*^*f/−*^*; Mvh*-Cre (Mvh-cKO) and *Ccnb1*^*f/−*^*; Stra8*-Cre (Stra8-cKO) mouse testes were significantly reduced compared with *Ccnb1*^*f/f*^ (Control) mice, suggesting that *Ccnb1* was efficiently deleted in male germ cells.

### Ablation of CCNB1 in gonocytes and spermatogonia led to sterility of male mice caused by germ cells’ depletion

**Figure 2:**
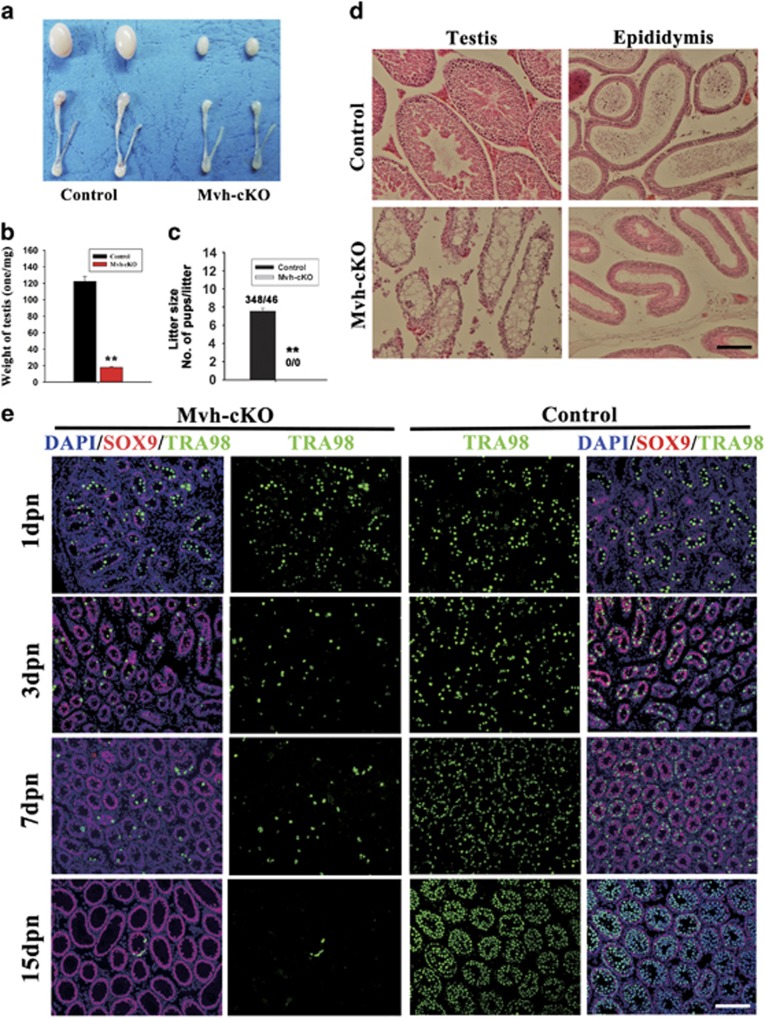
Deletion of Ccnb1 in early-stage male germ cells resulted in male mice sterile due to germ cells’ depletion. (**a**) Testis and epididymis of adult (2-month-old) control and Mvh-cKO mice. (**b**) Testis weight of 2-month-old Control and Mvh-cKO mice (*n*=12, Control; *n*=12, Mvh-cKO). (**c**) Litter size of female WT mice mated with Control and Mvh-cKO male mice, respectively. (**d**) Histological appearance of Mvh-cKO testis and epididymis. (**e**) Immunostaining of TRA98 and Sox9 in 1, 3, 7 and 15 dpn Mvh-cKO and Control littermate testis. TRA98, a marker of germ cell; Sox9, a marker of Sertoli cell. Bar=100 *μ*m

To inactivate the CCNB1 in male mouse germ cells at the early stage of germ cell development, we ablated CCNB1 in early stage of male germ cells using *Mvh-*Cre transgenic mice in which Cre recombinase was expressed in male germ cells at embryonic day 15 (E15).^30^ Adult Mvh-cKO male mice were overtly normal, but their testes were smaller than the control littermates (Figure 2a) and testis weight of the adult Mvh-cKO mice was strikingly reduced compared with the control littermates (Control: 122.2±5.963 mg; *n*=12; Mvh-cKO: 17.9±0.899 mg; *n*=12) (Figure 2b). Fertility testing showed that the Mvh-cKO male mice were completely sterile (Figure 2c). Histological analysis indicated that seminiferous tubules of Mvh-cKO mice were small and contained no germ cells (Figure 2d). The epididymis of Mvh-cKO mice were also smaller than the control littermates (Figure 1a) and no spermatozoa were observed (Figure 2d). To explore at which stage the germ cells disappeared in the Mvh-cKO mouse testis, we collected the day postnatal 1 (1 dpn), 3, 7 and 15 dpn control and Mvh-cKO mouse testes and examined the existence of germ cells by staining the germ cells with specific marker TRA98 and Sertoli cell marker SOX9. The results showed that quantity of germ cells in 1 dpn control and Mvh-cKO mouse testes had no difference, but germ cells notably reduced in 3 dpn Mvh-cKO mouse testis (Figure 2e). At 7 dpn, germ cells could proliferate normally in control mouse testes but not in Mvh-cKO mouse testes (Figure 2e). At 15 dpn, almost no germ cells existed in the seminiferous tubules of Mvh-cKO mice (Figure 2e). This phenotype may be caused by excessive apoptosis because there are no more spermatogonia. Germ cells were unable to detect at the 18 dpn Mvh-cKO mouse testis (Supplementary Figure S1A). We then investigated the expression of germline-specific genes (*Dazl*, *Oct4*, *Figla* and *Mvh*) in 7 and 120 dpn mouse testes using RT-PCR. We found that the expression of germline-specific genes significantly reduced in 7 dpn Mvh-cKO mice testis and were unable to detect in 120 dpn Mvh-cKO mice testis (Supplementary Figure S1B). Collectively, these results indicate that deletion of *Ccnb1* in early-stage male germ cells (gonocytes and spermatogonia) results in male sterile due to germ cells’ depletion before the first wave of spermatogenesis.

### Gonocytes and spermatogonia without CCNB1 were unable to proliferate normally and apoptosis was increased

**Figure 3:**
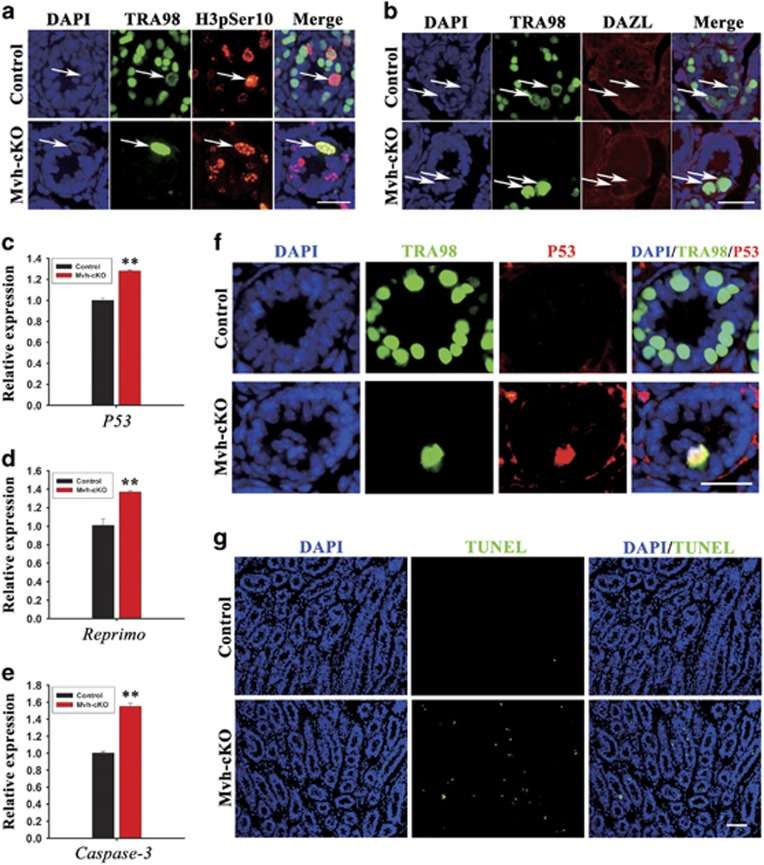
Germ cells’ depletion in Mvh-cKO mice is caused by germ cells’ apoptosis and inhibited proliferation. (**a**) Immunostaining of TRA98 and H3pSer10 in 7 dpn Control and Mvh-cKO mouse testes. (**b**) Immunostaining of TRA98 and Dazl in 7 dpn Control and Mvh-cKO mouse testes. TRA98 expressed in germ cells, primarily in nuclear in G1, S and G2 phase and in cytoplasm in M phase. Arrows indicate the germ cells in M phase. Dazl expressed in germ cells, primarily in cytoplasm in G1, S and G2 phase and in cytoplasm and nuclear region in M phase. In control mice, spermatogonia were able to complete nuclear envelop breakdown and chromosome condensation, but in Mvh-cKO mice, spermatogonia were unable to complete these processes. (**c**–**e**) Real-time PCR analysis of *p53*, *Reprimo* and *Caspase-3* in 7 dpn control and Mvh-cKO mouse testes. Three or more samples were used in each group in panels (**c**–**e**). (**f**) Immunostaining of TRA98 and p53 in 7 dpn Control and Mvh-cKO mouse testes. (**g**) Apoptosis signal significantly increased in 3 dpn Mvh-cKO mouse testes compared with control littermates detected by TUNEL analysis. Bar=100 *μ*m

To investigate what caused the germ cells depletion in Mvh-cKO male mice, we first examined proliferation of the germ cells in 7 dpn mouse testis. We found that, in the Mvh-cKO mice testis, although germ cells expressed M-phase-specific marker H3pSer10, they were unable to complete the M-phase events, such as chromosomes condensation and nuclear envelop breakdown (Figures 3a and b). We then examined the expression of several apoptosis-related genes in 7 dpn mouse testis by RT-PCR and QRT-PCR and found that the expression of *p53*, *Reprimo* and *Caspase-3* were notably increased in the Mvh-cKO mice testis (Supplementary Figure S2, Figures 3c–e). Moreover, immunostaining of p53 showed that some germ cells in 7 dpn Mvh-cKO mouse testes expressed the p53 protein, but no p53-active germ cell was observed in the control littermates (Figure 3f). In addition, TUNEL assays also showed that the apoptosis signal was remarkably increased in the Mvh-cKO mice testis compared with the control littermates (Figure 3g). These results suggest that male germ cells depletion in Mvh-cKO mouse testes is probably due to the inhibited proliferation of male germ cells and increased apoptosis.

### Ablation of CCNB1 in postnatal, premeiotic male germ cells does not have an effect on spermatocyte meiosis and male fertility

**Figure 4:**
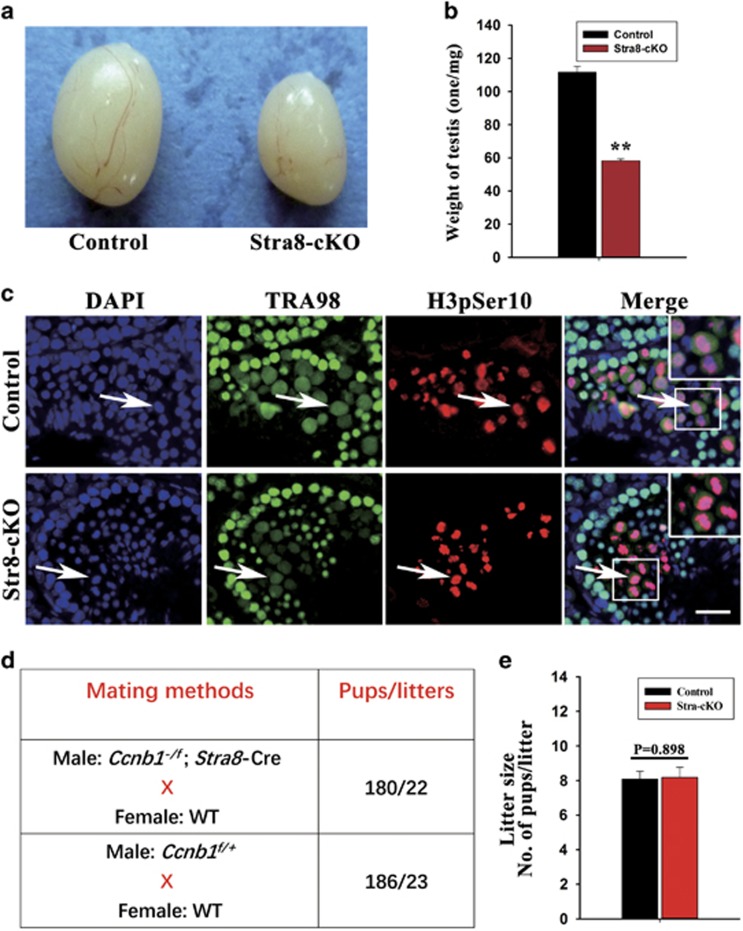
Deletion of Ccnb1 in postnatal, premeiotic germ cells has no effect on spermatocyte meiosis and male fertility. (**a**) Testis of 2-month-old Control and Mvh-cKO mice. (**b**) Testis weight of 2-month-old Control and Stra8-cKO mice (*n*=12, Control; *n*=12, Stra8-cKO). (**c**) Immunostaining of TRA98 and H3pSer10 in 3-month-old Control and Stra8-cKO mice testis. (**d**) Fertility analysis: mating methods and pubs/litters obtained. (**e**) Litter size of female WT mice mated with Control and Stra8-cKO male mice, respectively. Bar=100 *μ*m

Because male germ cells were completely disrupted before the first wave of spermatogenesis in Mvh-cKO mice, the function of CCNB1 in meiosis of spermatocytes remain unclear. To investigate the role of CCNB1 in meiosis of mammalian male germ cells, we generated the Stra8-cKO mice. In these mice, *Ccnb1* floxed allele was deleted by Stra8-Cre in postnatal, premeiotic male germ cells.^31^ Testes of adult Stra8-cKO mice were smaller than control mice (Figure 4a) and testis weight is markedly reduced (Control: 111.6±3.602 mg, *n*=12; Stra-cKO: 58.25±1.115 mg, *n*=12; Figure 4b). We then examined the process of male germ cells meiosis and found that meiosis of spermatocytes is normal in Stra8-cKO mice. The spermatocytes in Stra8-cKO mouse testis expressed the M-phase-specific marker H3pSer10, could complete nuclear envelop breakdown and chromosome condensation normally and generate haploid spermatids (Figure 4c). In addition, adult Stra8-cKO male mice were fertile and the fertility were normal compared with control littermates (Figures 4d and e).The hypoplasia testis of Stra8-cKO mice is probably due to the defects of germ cell mitosis but not of the germ cell meiosis, as Stra8-Cre can also delete *Ccnb1* floxed allele in part of undifferentiated spermatogonia. We then examined the early-stage germ cells by immunostaining of the germ cell-specific marker TRA98 in 15 dpn Stra8-cKO mouse testes and found that the germ cells were notably reduced (Supplementary Figure S3), suggesting that the hypoplasia testis of Stra8-cKO mice were indeed due to the inhibited proliferation of early-stage germ cells. Collectively, these results indicate that ablation of CCNB1 in premeiotic male germ cells using Stra8-Cre results in hypoplasia testis but does not have an effect on meiosis of spermatocytes and male fertility, suggesting that CCNB1 may be redundant in meiosis of spermatocytes.

### Ablation of CCNB1 in male germ cells might promote their differentiation by downregulating the expression of Lin28a and upregulating the expression of let-7 miRNAs

**Figure 5:**
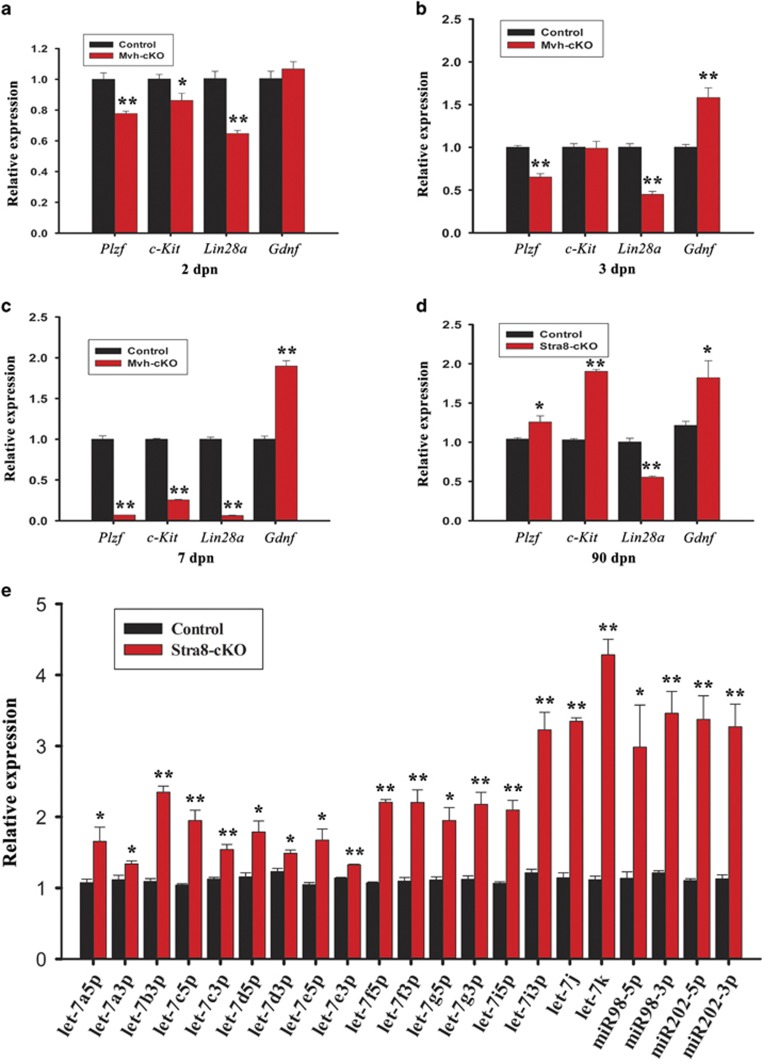
Deletion of Ccnb1 in germ cells promoted their differentiation and Lin28a/let-7 axis involved in this process. (**a**) Real-time PCR analysis of *Plzf*, *c-Kit*, *Lin28a* and *Gdnf* expression in 2 dpn control and Mvh-cKO mice testis. (**b**) Real-time PCR analysis of *Plzf*, *c-Kit*, *Lin28a* and *Gdnf* expression in 3 dpn control and Mvh-cKO mice testis. (**c**) Real-time PCR analysis of *Plzf*, *c-Kit*, *Lin28a* and *Gdnf* expression in 7 dpn control and Mvh-cKO mice testis. (**d**) Real-time PCR analysis of *Plzf*, *c-Kit*, *Lin28a* and *Gdnf* expression in 90 dpn control and Stra8-cKO mice testis. (**e**) Real-time PCR analysis of let-7 family miRNAs expression in 90 dpn Control and Stra8-cKO mice testis. In panels (**a**–**e**), ≥3 samples were used in each group in qPCR

CCNB1 may not have an essential role in spermatogonial germ cells’ differentiation, as Stra8-cKO mice have normal fertility. To investigate the effect of CCNB1 ablation on spermatogonial germ cells’ differentiation, we examined the expression of the gonocytes and undifferentiated spermatogonia-specific genes, *Plzf* and *Lin28a* and a differentiated spermatogonia-specific gene *c-Kit* in 2, 3 and 7 dpn Mvh-cKO and adult Stra8-cKO mouse testis.^32, 33, 34, 35, 36, 37, 38, 39, 40^ The expression of *c-Kit* is significantly reduced in 2 dpn Mvh-cKO mice testis (Figure 5a), but in 3 dpn mice testis, its expression had returned to normal level (Figure 5b). Moreover, the expression of *c-Kit* in adult Stra8-cKO mice was strikingly increased (Figure 5d). These observations indicate that ablation of CCNB1 in gonocytes and spermatogonia does not inhibit their differentiation; in contrast, it might promote their differentiation. Interestingly, we observed that, in contrast to 2, 3 and 7 dpn Mvh-cKO mouse testis, the expression of Plzf in adult Stra8-cKO mouse testis was significantly increased (Figures 5a–d). The best interpretation of this phenomenon is that *Ccnb1* is not completely depleted in all spermatogonia in Sta8-cKO male mice, as Stra8-Cre just delete *Ccnb1* in part of undifferentiated spermatogonia and in all differentiated spermatognia.^31^ We also found that the expression of *Gdnf*, which can promote self-renewal of undifferentiated spermatogonia,^41^ was remarkably increased in the testis of 3 and 7 dpn Mvh-cKO mice and adult Stra8-cKO mice testis (Figures 5b–d). However, even with the higher expression of *Gdnf*, the expression of *Plzf* was still notably reduced in 3 and 7 dpn Mvh-cKO mice testis (Figures 3b and c), suggesting that CCNB1 is critically required for the undifferentiated germ cells’ self-renewal. Another interesting phenomenon is that, in contrast to *Plzf*, the expression of *Lin 28a* was notably reduced in the adult Stra8-cKO mice testis. In fact, although *Plzf* and *Lin28a* were remarkably reduced in 2, 3 and 7 dpn Mvh-cKO mice testis, the expression of *Lin28a* reduced more severely than *Plzf*. These results indicate that LIN28A may be involved in germ cells’ differentiation. LIN28A specifically regulates the maturation of let-7 miRNAs.^42, 43, 44^ We then investigated the expression of let-7 miRNAs in the adult Stra8-cKO mouse testes and found that let-7 miRNAs were remarkably increased. Collectively, these results indicate that ablation of CCNB1 might promote the undifferentiated germ cells’ differentiation by downregulating the expression of *Lin28a* and upregulating the expression of let-7 miRNAs.

## Discussion

In the present study, we reported the requirement of CCNB1 in spermatogenesis using conditional knockout mice lacking CCNB1 in male germ cells. Ablation of CCNB1 in mouse gonocytes and spermatogonia results in male sterile due to germ cells’ depletion, whereas ablation of CCNB1 in postnatal, premeiotic male germ cells does not have an effect on the meiosis of spermatocyte and male fertility. We found that germ cells’ depletion in Mvh-cKO mice is probably due to inhibited proliferation and increased apoptosis. We also showed that ablation of CCNB1 in undifferentiated spermatogonia might promote their differentiation by downregulating the expression of *Lin28a* and upregulating the expression of let-7 miRNAs. These results indicate that CCNB1 is critically required for the proliferation of gonocytes and spermatogonia but may be redundant in spermatocytes. Previous studies showed that CDK1 is essential for mitosis and meiosis in mice. Mammalian somatic cells without CDK1 are unable to complete mitosis while mammalian oocytes or spermatocytes without CDK1 are unable to complete meiosis.^45, 46, 47, 48^ In the present study, we show that CCNB1, one regulatory subunit of CDK1, is essential for the mitosis of early-stage male germ cells but may be redundant in meiosis of male germ cells.

The mitosis of male germ cells involves the proliferation of primordial germ cells (PGCs), gonocytes and spermatogonia. The *Mvh-*Cre has its role beginning from E15, and at 1 dpn recombinase efficiency can reach >95%.^30^ Mvh-Cre can delete genes specifically in gonocytes, spermatogonia, spermatocytes and spermatids but is unable to delete genes in PGCs. In this study, the early stage of male germ cells refers to gonocytes and spermatogonia ranging from 1 to 15 dpn. Ablation of CCNB1 in these cells results in male germ cells’ depletion caused by inhibited proliferation and increased apoptosis. However, it is unclear whether CCNB1 is required for the proliferation of PGCs. To study the requirement for CCNB1 function in PGCs, an earlier expressed germ cell-specific Cre may be needed.

The first wave of spermatogenesis is different with the adult waves of spermatogenesis. In Mvh-cKO mouse testes, germ cells were completely depleted before the first wave of spermatogenesis. Therefore, it is unclear whether CCNB1 is essential for the spermatogonia in adult male mice. To test the requirement of CCNB1 in spermatogonia in adult male mice, we also deleted CCNB1 in undifferentiated spermatogonia using Ngn3-Cre.^49^ We found that the male *Ccnb1* ^*f/−*^*; Ngn3*-Cre (Ngn3-cKO) mice are subfertile; they can generate a little mount of spermatozoa (Supplementary Figure S4E). The testes of Ngn3-cKO mice are smaller than control and almost no spermatozoa exist in epididymis (Supplementary Figures S4A–C). The expression of *Ccnb1*, *Mvh*, *Stra8*, *Sycp3* and *Prm1* were notably reduced in Ngn3-cKO adult mouse testes (Supplementary Figure S4D), whereas the expression of *Plzf*, *Gfra1*, *c-kit*, *p53*, *Reprimo*, *Caspase-3* and *Gdnf* were remarkably increased in Ngn3-cKO adult mouse testis compared with control littermates (Supplementary Figure S4D). The phenotype of Ngn3-cKO mice are more severe than Stra8-cKO mice but less than Mvh-cKO mice. The defects of Mvh-cKO and Ngn3-cKO mice indicate that CCNB1 is critically required for the proliferation of gonocytes and spermatogonia in pubertal mouse testes and for the proliferation of spermatogonia in adult mouse testes.

GDNF, secreted by Sertoli cells, is a factor essential for undifferentiated spermatogonia self-renewal.^41^ We found that expression of *Gdnf* was normal in 2 dpn Mvh-cKO mouse testes but significantly increased in 3 and 7 dpn Mvh-cKO and adult Stra8-cKO and Ngn3-cKO mouse testes compared with control littermates. These results suggest that ablation of CCNB1 in germ cells does not inhibit the secretion of GDNF; in contrast, the expression of Gdnf is increased. Consisted with the high expression of *Gdnf* in Stra8-cKO and Ngn3-cKO mice testis, the expression of *Plzf* was remarkably increased, indicating that high expression of *Gdnf* may promote the self-renewal of undifferentiated spermatogonia. However, the higher expression of *Gdnf* in 3 or 7 dpn Mvh-cKO mouse testes was unable to promote the proliferation of gonocytes or spermatogonia. These observations indicate that CCNB1 is critically required for the proliferation of gonocytes and spermatogonia. Even when high GDNF exists but not CCNB1, the gonocytes and spermatogonia are unable to proliferate normally.

The expression of *Plzf* and *c-Kit* were notably increased in adult Stra8-cKO and Ngn3-cKO mouse testis compared with control littermates, whereas in Mvh-cKO mouse testis these two genes were markedly reduced. The best interpretation of this phenomenon is that CCNB1 was not ablated in all spermatogonia in Stra8-cKO and Ngn3-cKO mice, and the spermatogonia with normal level of CCNB1 could proliferate normally. Moreover, high level of GDNF in Stra8-cKO and Ngn3-cKO mouse testes further promote the proliferation of these CCNB1-remaining spermatogonia. In short, CCNB1 was not completely deleted in all spermatogonia in Stra8-cKO and Ngn3-cKO mice, in these mice spermatogonial stem cell (SSC) pool still exist, SSCs can self-renew; however, in Mvh-cKO mice, CCNB1 was ablated in all spermatogonia and SSC pool was unable to maintain and finally led to germ cells’ depletion and male sterility (Supplementary Figure S5).

We also showed that germ cells’ depletion in male Mvh-cKO mice is due to inhibited proliferation and increased apoptosis in germ cells. However, ablation of CCNB1 might not inhibit spermatogonial differentiation; in contrast, it may promote spermatogonial differentiation. LIN28A is a marker of undifferentiated spermatognia and highly expressed in undifferentiated spermatognia in mice testis.^34, 35, 37^ LIN28A mainly represses the maturation of let-7 miRNAs, whereas the maturation of other miRNAs is largely unaffected by LIN28A.^42, 43, 44^ Because the mRNAs of *Lin28a* are themselves let-7 targets, this LIN28A/let-7 axis creates a double-negative feedback loop whereby either let-7 or LIN28A is expressed at high levels, generating a differentiated or an embryonic cell fate, respectively.^50^ Previous studies showed that LIN28A/let-7 axis regulates proliferation and differentiation of PGCs and spermatogonial progenitor cyclic expansion.^34, 36, 37^ In the present study, we found that, in contrast to higher expression of *Plzf*, the expression of *Lin28a* was remarkably reduced in adult Stra8-cKO mouse testis. Moreover, though the expression of *Plzf* and *Lin28a* were notably reduced in 2, 3 and 7 dpn Mvh-cKO mouse testis, *Lin28a* reduced more severely than *Plzf*. In addition, we also found that the mature miRNA level of let-7 family were notably increased in adult Stra8-cKO mice testis. These results indicate that high level of c-Kit expression in Stra8-cKO mice testes is probably due to the change of LIN28A/let-7 axis, with low level of *Lin28a* and high level of let-7 promoting the differentiation of spermatogonia.

Stra8-cKO mice have normal fertility, albeit their testis is smaller than control, suggesting that CCNB1 may be redundant in meiosis of spermatocytes. CCNB2, which is also highly expressed in testis, may compensate the loss of CCNB1 in mutant mice. To investigate whether CCNB1 is compensated by CCNB2 in meiosis of spermatocytes, we intend to generate *Ccnb2*^*−/−*^*; Ccnb1*^*f/f*^*; Stra8*-Cre mice. We found that the fertility of male *Ccnb2*^*−/+*^*; Ccnb1*^*f/f*^*; Stra8*-Cre mice is remarkably reduced. When we crossed male *Ccnb2*^*−/+*^*; Ccnb1*^*f/f*^*; Stra8*-Cre mice with female *Ccnb2*^*−/+*^*; Ccnb1*^*f/f*^*; Stra8*-Cre or *Ccnb2*^*−/+*^*; Ccnb1*^*f/f*^ mice for 3 months, only one or three pups were born. Until now, the *Ccnb*^*−/−*^*; Ccnb1*^*f/f*^*; Stra8*-cKO male mice have not been produced. The observations suggest that CCNB2 might compensate the role of CCNB1 in meiosis of spermatocytes in mutant mice.

In summary, CCNB1 is critically required for the spermatogenesis. Ablation of CCNB1 in gonocytes and spermatogonia rendered them unable to proliferate normally and apoptosis increased, finally resulting in germ cells’ depletion and male sterility. However, ablation of CCNB1 in premeiotic male germ cells did not have effect on meiosis of spermatocytes and male fertility, suggesting that CCNB1 may be redundant in meiosis of spermatocytes, and other cyclins such as CCNB2 may compensate the role of CCNB1 in meiosis of spermatocytes in mutant mice. We also found that ablation of CCNB1 in spermatogonia may promote their differentiation by downregulating *Lin28a* and upregulating let-7 miRNAs. Our study is the first to prove *in vivo* the essential role of CCNB1 in mammalian spermatogenesis. To better understand and cure some cell cycle abnormal diseases such as cancer, it is important to understand fully the function of CCNB1 *in vivo*.

## Materials and methods

### Animals

*Ccnb1* targeting ES cells were obtained from the European Conditional Mouse Mutagenesis Program. *Ccnb1* floxed mice were generated with the standard procedure. Genotypes were identified by PCR analysis (F: 5′-CAAGCACTTTACCACCGAACTAT-3′ R: 5′-GTCAGAAGACAGCTACTGTGTAC-3′). The wild-type allele generated a band at 673 bp, while the floxed allele was at 475 bp and cKO allele could not generate band by using these primers. The mice obtained were from mixed backgrounds of 129 and C57BL/6J. Mvh-Cre (The Jackson Laboratory, Bar Harbor, ME, USA, stock no. 006954) and Stra8-Cre (The Jackson Laboratory, stock no. 008208) mice were used in the present study and were described previously.^30, 31^ All animals were kept in accordance with the protocols approved by the guidelines of the Institutional Animal Care and Use Committee of the Institute of Zoology (IOZ), Chinese Academy of Sciences (CAS), Beijing, China.

### qRT-PCR and RT-PCR analysis

Total RNA was isolated from testes using Trizol (TIANGEN, Beijing, China) according to the manufacturer’s protocol. The RNA was reverse-transcribed with M-MLV reverse transcriptase (TIANGEN, Beijing, China) and qPCR with GoTaq qPCR Master Mix (Promega, Wisconsin, USA, A6001/2) according to the manufacturer’s protocols. Total Micro RNA was isolated from testes using the miRcute miRNA Isolation Kit (DP501, TIANGEN), reverse-transcribed with miRcute miRNA First-Strand cDNA Synthesis Kit (KR201, TIANGEN) and qPCR with miRcute Plus miRNA qPCR Detection Kit (SYBR Green) (FP411, TIANGEN) following the manufacturer’s instructions. The Ct values were normalized to the internal control (GAPDH for RNA and U6 for micro-RNA) and presented as a relative expression level. All primers for qRT-PCR and RT-PCR are described in Supplementary Tables S1 and S2, respectively.

### Western blotting analysis

Proteins of testis were separated on 10% SDS-PAGE gels and transferred to PVDF membranes and probed with primary antibodies as follows: CCNB1 (Abcam, Cambridge, UK, ab72, 1 : 500); GAPDH (Bioworld, Minnesota, USA, MB001, 1 :5000) and followed with secondary antibodies conjugated to horseradish peroxidase (ZSGB-BIO, Beijing, China) at a dilution of 1 : 5000 and detected by the ECL System (Pierce, Waltham, MA USA).

### Tissue collection and histological examination

Mouse testes and epididymis were collected and weighed, fixed in 4% paraformaldehyde and then embedded in paraffin. Five sections of each testis and epididymis (5 *μ*m, taken 200 *μ*m apart) were stained with hematoxylin–eosin for normal histological analysis.

Immunohistochemistry (IHC) staining was performed with the standard procedure, using horseradish peroxidase-conjugated anti-IgG secondary antibodies (Jackson ImmunoResearch, West Grove, PA, USA), visualized with 3, 3′-diaminobenzidine. The slides were counterstained with hematoxylin.

For immunofluorescence (IF) staining, tissue sections were dewaxed and rehydrated and then antigen retrieved in 10 mM sodium citrate buffer. Sections were blocked (5% BSA) and incubated with primary antibodies at 4 °C overnight. Sections were washed and incubated with FITC or TRITC-conjugated secondary antibodies (1 : 200; Jackson ImmunoResearch) for 1 h at room temperature and 4′, 6-diamidino-2-phenylindole (DAPI) (Sigma-Aldrich, St. Louis, MO, USA) was used to visualize the nucleus. The antibodies used for the IHC or IF experiment were: anti-Wt1 antibody (Santa Cruz, St. Louis, MO, USA, sc-7358, 1 : 100), anti-TRA98 antibody (Abcam, ab82527, 1 : 200), anti-SOX9 antibody (Cell Signaling Technology, Danvers, MA, USA, 2524S, 1 : 100), anti-H3pSer10 antibody (Merck Millipore, Darmstadt, Germany, 06-570, 1 : 100), and anti-DAZL antibody (Abcam, ab34139, 1 : 100).

### Apoptosis assay

Cell apoptosis was evaluated in the testicular sections using a terminal deoxynucleotidyltransferase-mediated dUTP nick end labeling (TUNEL) assay, using commercial kit (Promega) following the manufacturer’s instruction. DAPI was used to visualize the nucleus.

### Fertility testing

To test the fertility of the male mice, six 8-week-old control and six 8-week-old cKO mice were mated with wild-type proven fertility female C57 mice in a ratio of 1 : 2. Successful conception was defined by the presence of vaginal plug and subsequent visibly growing abdomen. The pregnant females were then separated and the litter sizes were recorded after birth.

### Statistical analysis

All experiments were performed at least in triplicate and the results were presented as mean±S.E.M. Two groups were compared by using Student’s *t-*test and *P*<0.05 (*) and *P*<0.01 (**) were considered significant and very significant, respectively.
