# Supplementary figures and images for: Requirement for CCNB1 in mouse spermatogenesis
Source: Cell Death Dis. 2017 Oct 26;8(10):e3142–. doi: 10.1038/cddis.2017.555 (PMC5680922; doi:10.1038/cddis.2017.555)

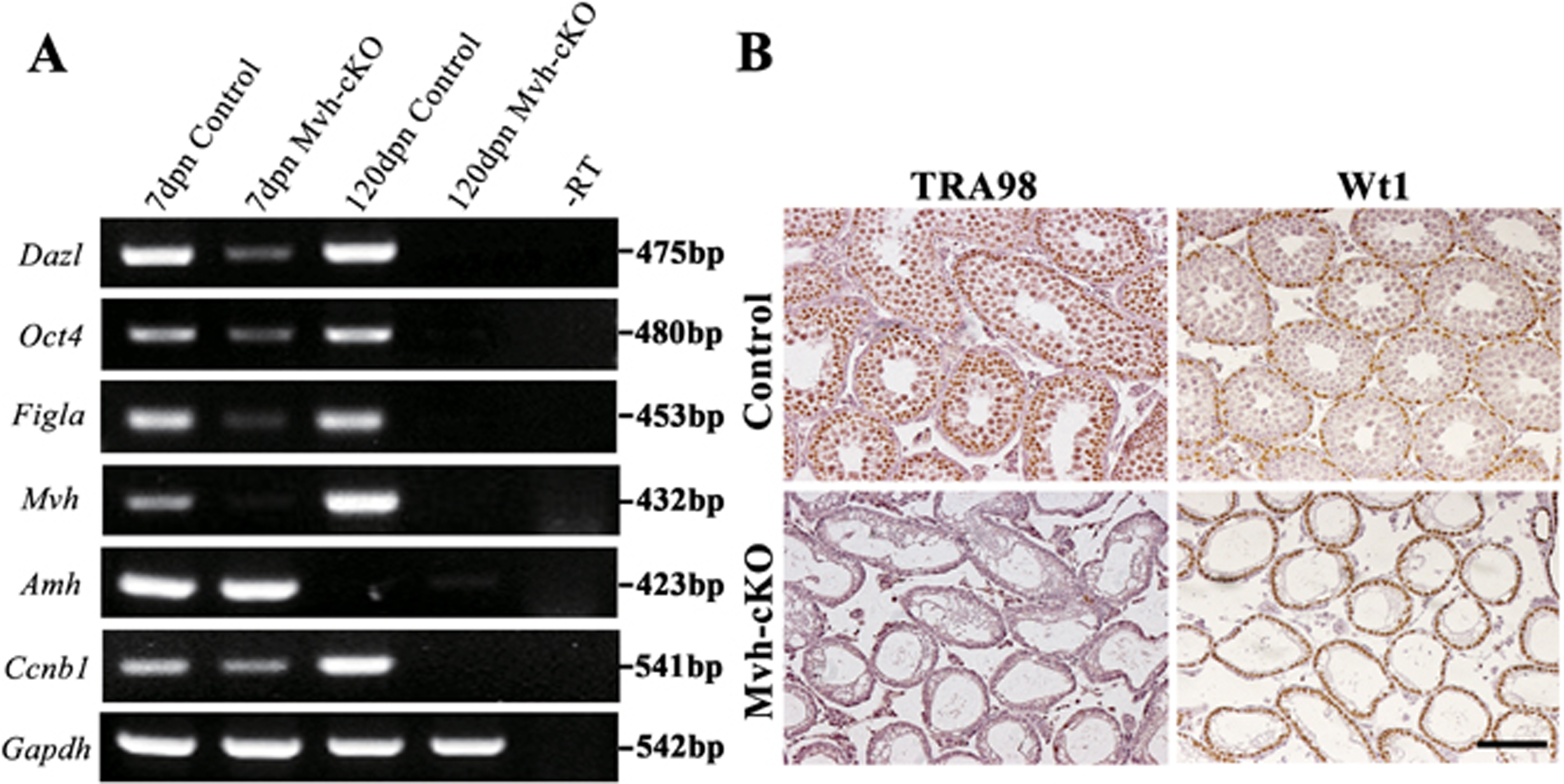

Supplement: Supplementary Figure 1 [file cddis2017555x1.tif]

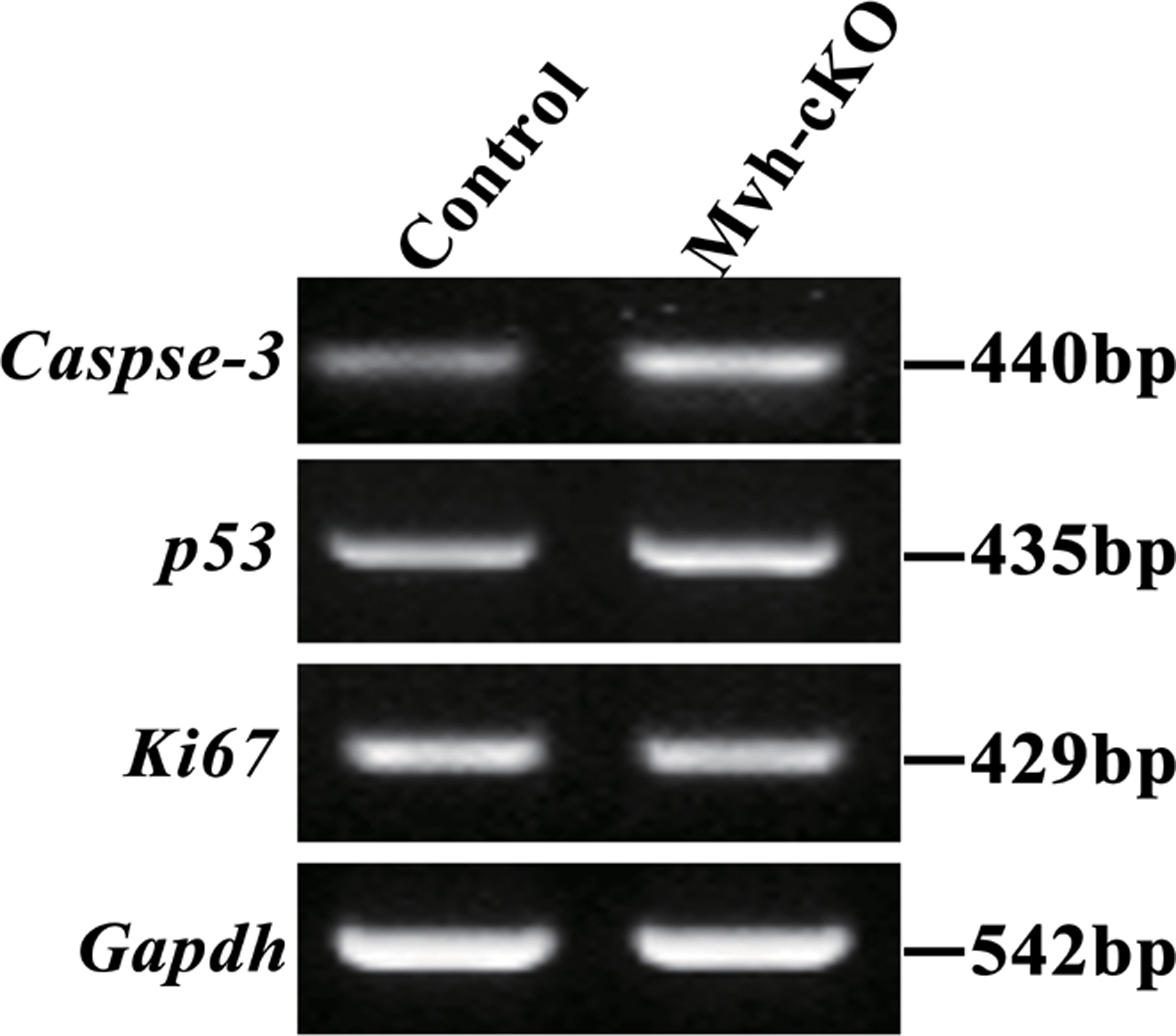

Supplement: Supplementary Figure 2 [file cddis2017555x2.tif]

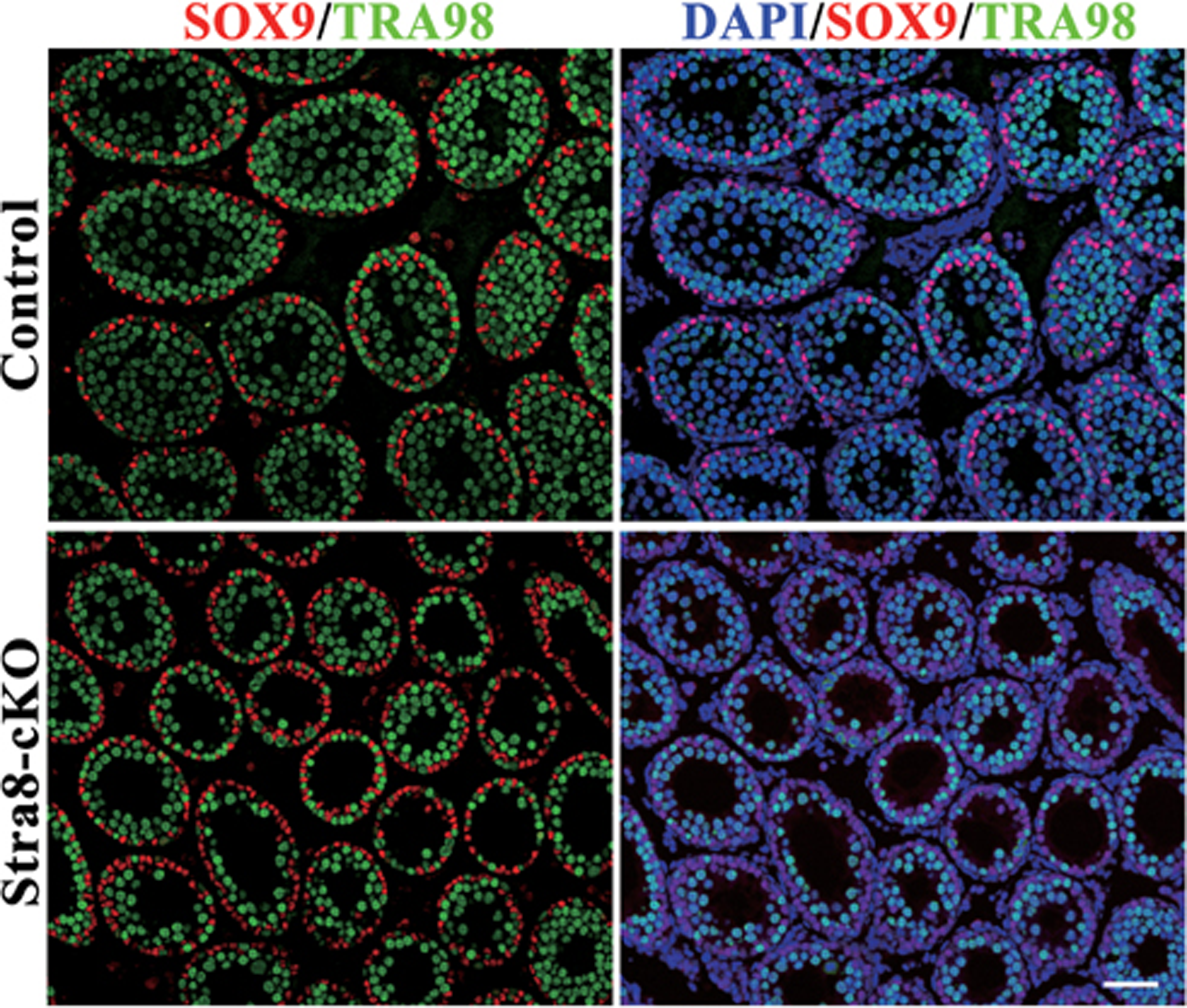

Supplement: Supplementary Figure 3 [file cddis2017555x3.tif]

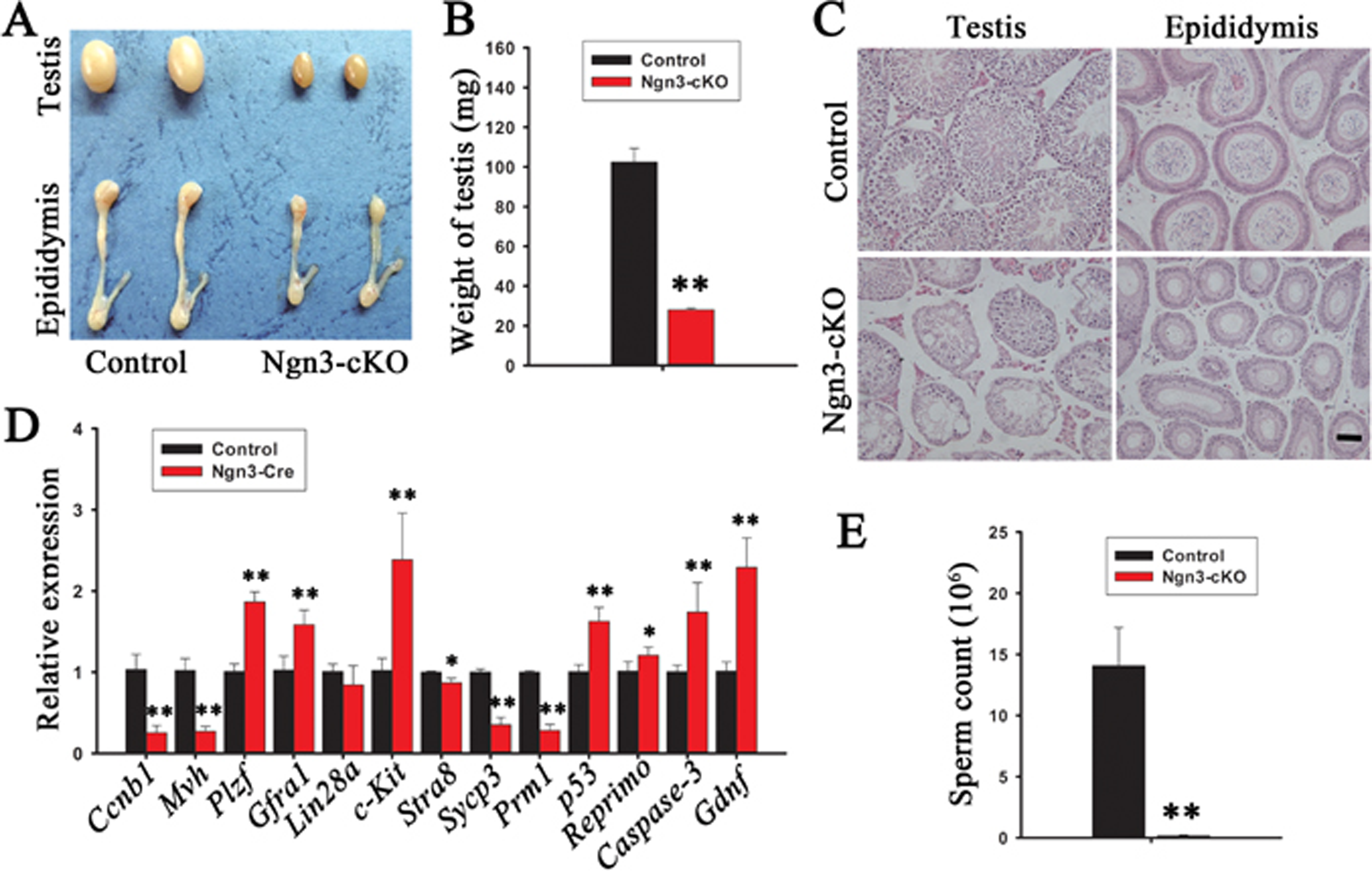

Supplement: Supplementary Figure 4 [file cddis2017555x4.tif]

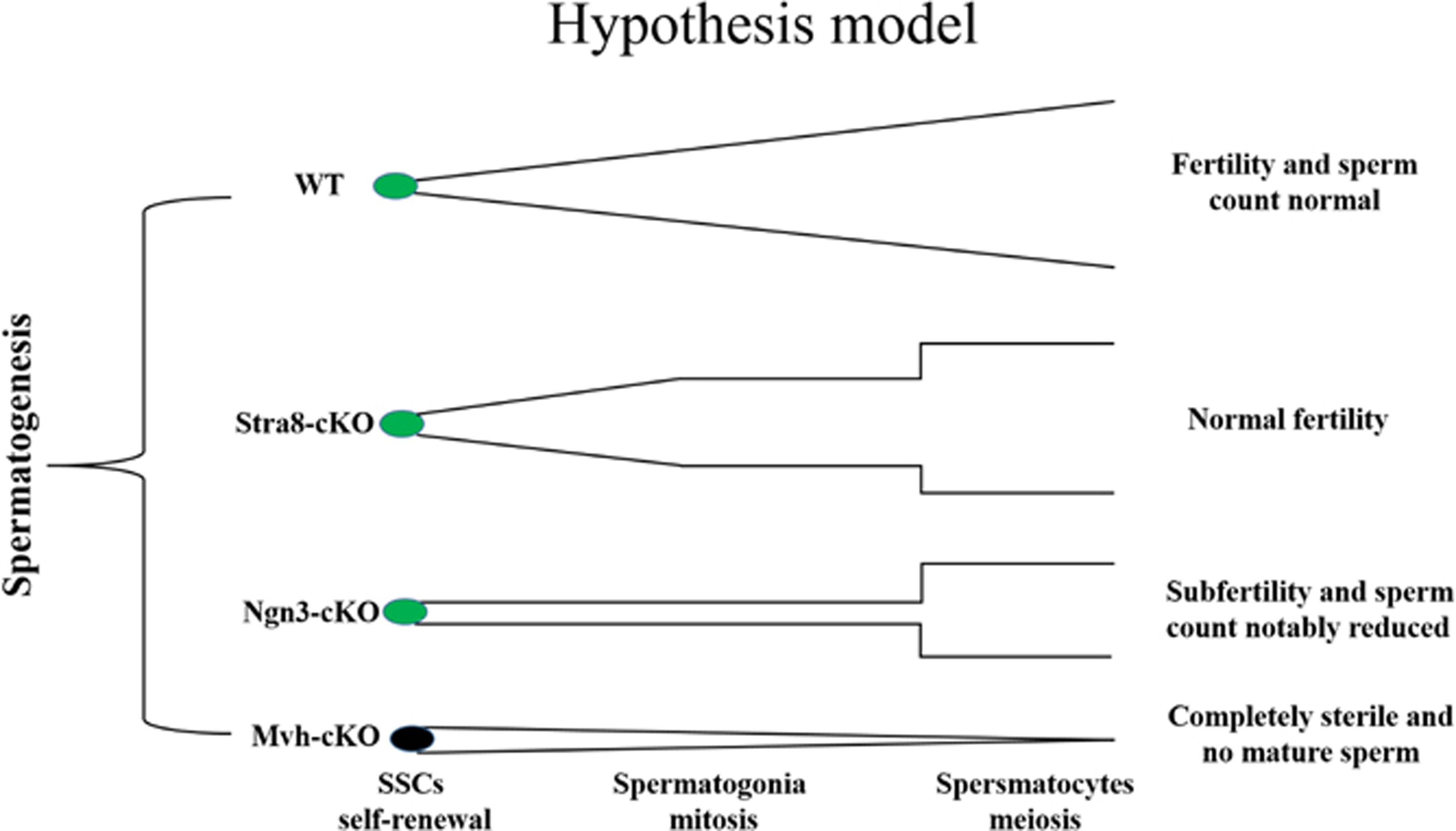

Supplement: Supplementary Figure 5 [file cddis2017555x5.tif]
