# Supplementary material for: Requirement for CCNB1 in mouse spermatogenesis
Source: Cell Death Dis. 2017 Oct 26;8(10):e3142–. doi: 10.1038/cddis.2017.555 (PMC5680922; doi:10.1038/cddis.2017.555)
Supplement: Supplementary Figure Legends and Tables [file cddis2017555x6.doc]

**Supporting Information**

Fig. S1. Deletion of Ccnb1 in early stage of germ cells resulted in germ cell depletion. (A) RT-PCR analysis of *Dazl*, *Oct4*, *Figla*, *Mvh*, *Amh* and *Ccnb1* in 7dpn and 120dpn Control and Mvh-cKO mice testis. Germ cell-specific genes (*Dazl*, *Oct4*, *Figla* and *Mvh*) were remarkably reduced in 7dpn Mvh-cKO mice testis compared with control littermates and no germ cell-specific genes were detected in 120dpn Mvh-cKO mice testis. (B) Immunostaining of TRA98 and Wt1 in 18dpn Control and Mvh-cKO mice testis. In 18dpn Mvh-cKO mice seminiferous tubules, no germ cells was found and only Sertoli cell remained.

Fig. S2. RT-PCR analysis of *Caspase-3*, *p53* and *Ki67* in 7dpn Control and Mvh-cKO mouse testes.

Fig. S3. Hypoplasia testis of Stra8-cKO mice are due to germ cell mitosis defects.

Immunostaining of TRA98 and Sox9 in 15dpn Control and Stra8-cKO mice testis. In Stra8-cKO testis, germ cells were remarkably reduced compared with control littermates.

Fig. S4. Ablation of CCNB1 in undifferentiated spermatogonia using Ngn3-Cre. (A) Testis and epididymis of 2-month-old *Ccnb1^f/+^* (Control) and *Ccnb1^f/-^; Ngn3*-Cre (Ngn3-cKO) mice. (B) Testis weight of 2-month-old Control and Ngn3-cKO mice (n=6, Control; n=6, Mvh-cKO). (C) Histological appearance of Control and Ngn3-cKO testis and epididymis. (D) Real-time PCR analysis of *Ccnb1*, Mvh, Plzf, Gfra1, Lin28a, c-Kit, Stra8, Sycp3, Prm1, p53, *Reprimo*, *Caspase-3* and Gdnf in 2-month-old control and Mvh-cKO mice testis. (E) Sperm count of 2-month-old Control and Ngn3-cKO. Sperm were collected from the caudal epididymidis and counted using hemocytometer (Control: (14.067±3.12)×10^6^, n=3; Ngn3-cKO: (0.154±0.07)×10^6^, n=3). Bar=100μm.

Fig. S5. Hypothesis model. In wild type (WT) male mice, SSCs is normal and can produce amounts of spermatozoa. In Mvh-cKO mice, CCNB1 is deleted in all SSCs, SSCs cannot self-renewal led to male sterile caused by male germ cells depletion. In Ngn3-cKO mice, CCNB1 is not deleted in all SSCs; SSCs can self-renewal and generate a little amount of spermatozoa, resulting in male subfertility. In Stra8-cKO mice, CCNB1 was deleted only in a little part of undifferentiated spermatogonia, SSCs can self-renewal and generate amounts of spermatozoa led to male mice have normal fertility. Ablation of CCNB1 in spermatogonia inhibits their proliferation but not their differentiation, in contrast, CCNB1 deletion promote spermatogonia differentiation. CCNB1 may redundant for meiosis of spermatocytes, as both Stra8-cKO and Ngn3-cKO mice can complete meiosis normally and generate functional spermatozoa.

**Table S1: Primers for qRT-PCR.**

| **Primers for qRT-PCR** | | | |
| --- | --- | --- | --- |
| **Gene name** | **Forward primers** | **Reverse primers** | **Size (bp)** |
| *Gapdh* | GGAGAAACCTGCCAAGTATG | GGAGAAACCTGCCAAGTATG | 112 |
| *Ccnb1* | GAGCTATCCTCATTGACTGG | CATCTTCTTGGGCACACAAC | 125 |
| *p53* | CTCTGAGTATACCACCATCC | CACGAACCTCAAAGCTGTCC | 113 |
| *Reprimo* | GTGTGGTGCAGATCGCAGT | ATCATGCCTTCGGACTTGATG | 100 |
| *Caspase-3* | CATACATGGGAGCAAGTCAG | CCATGAATGTCTCTCTGAGG | 127 |
| *Plzf* | CCACCTTCGCTCACATACAG | CTTGTGGCTCTTGAGTGTGC | 136 |
| *c-Kit* | GACGGTACATGGCTGCATTC | GTGACTTGTTTCAGGCACAG | 123 |
| *Lin28a* | GGCATCTGTAAGTGGTTCAACG | GCCAGTGACACGGATGGATT | 210 |
| *Gdnf* | TAATGTCCAACTGGGGGTCT | CGCTTCGAGAAGCCTCTTAC | 145 |
| mmu-let-7a-5p | TGAGGTAGTAGGTTGTATAGTT | | |
| mmu-let-7a-1-3p | CTATACAATCTACTGTCTTTCC | | |
| mmu-let-7b-3p | TGAGGTAGTAGGTTGTGTGGTT | | |
| mmu-let-7c-5p | TGAGGTAGTAGGTTGTATGGTT | | |
| mmu-let-7c-1-3p | CTGTACAACCTTCTAGCTTTCC | | |
| mmu-let-7d-5p | AGAGGTAGTAGGTTGCATAGTT | | |
| mmu-let-7d-3p | CTATACGACCTGCTGCCTTTCT | | |
| mmu-let-7e-5p | TGAGGTAGGAGGTTGTATAGTT | | |
| mmu-let-7e-3p | TATACGGCCTCCTAGCTTTCC | | |
| mmu-let-7f-5p | TGAGGTAGTAGATTGTATAGTT | | |
| mmu-let-7f-2-3p | CTATACAGTCTACTGTCTTTC | | |
| mmu-let-7g-5p | TGAGGTAGTAGTTTGTACAGTT | | |
| mmu-let-7g-3p | ACTGTACAGGCCACTGCCTTGC | | |
| mmu-let-7i-5p | TGAGGTAGTAGTTTGTGCTGTT | | |
| mmu-let-7i-3p | CTGCGCAAGCTACTGCCTTGCT | | |
| mmu-let-7j | TGAGGTATTAGTTTGTGCTGTTAT | | |
| mmu-let-7k | TGAGGTAGGAGGTTGTGTG | | |
| mmu-miR-98-5p | TGAGGTAGTAAGTTGTATTGTT | | |
| mmu-miR-98-3p | CTATACAACTTACTACTTTCCT | | |
| mmu-miR-202-5p | TTCCTATGCATATACTTCTTT | | |
| mmu-miR-202-3p | AGAGGTATAGCGCATGGGAAGA | | |

Table S2: Primers for RT-PCR

| **Primers for RT-PCR:** | | | |
| --- | --- | --- | --- |
| **Gene name** | **Forward primers** | **Reverse primers** | **Size (bp)** |
| *GAPDH* | ATGGTGAAGGTCGGTGTGAA | GCAGTGATGGCATGGACTGT | 542 |
| *Ki67* | GCGATGGCGTCCTCGGCTCACCT | GCGTGAAGCTTTGGTATCTTGAC | 429 |
| *p53* | GCGATGACTGCCATGGAGGAGTC | GCGGCTGACCCACAACTGCACAG | 435 |
| *Caspase-3* | GCGATGGAGAACAACAAAACCTC | GCGTAGTCGCCTCTGAAGAAGCT | 440 |
| *Dazl* | GTGTGTCGAAGGGCTATGGAT | ACAGGCAGCTGATATCCAGTG | 475 |
| *Oct4* | AGCTGCTGAAGCAGAAGAGG | GGTTCTCATTGTTGTCGGCT | 480 |
| *Figla* | CCAAAGAGCGTGAACGGATAA | TCTTCCAGAACACAGCCGAGT | 453 |
| *Mvh* | GCGATGGGAGATGAAGATTGGGA | GCGGCCTGATGCTTCTGAATCGT | 432 |
| *Amh* | GCGATGCAGGGGCCACACCTCTC | GCGGAGCTCGGGCTCCCATATCA | 423 |
| *Ccnb1* | GAAGAGCAGTCAGTTAGACC | GTGTCCATTCACCGTTGTCA | 541 |
